# Challenges faced by Latin America health systems

**DOI:** 10.1590/0102-311XEN233225

**Published:** 2026-08-10

**Authors:** Leonardo Castro

**Affiliations:** 1 Escola Nacional de Saúde Pública Sergio Arouca, Fundação Oswaldo Cruz, Fiocruz, Rio de Janeiro, Brasil.

This article proposes a comprehensive reflection, both panoramic and synthetic, on the current challenges faced by Latin America health systems. This is not a simple task as the moment is extremely uncertain regarding the near future. Climate emergency and the risk of new pandemics are in themselves immense challenges for health systems. In addition to the disruption of ecosystems and loss of biodiversity, with its disastrous consequences, there are democratic setbacks, increased inequalities, wars, and the intensification of geopolitical conflicts. Shortly after COVID-19, the political and economic conditions that favor pandemics not only continue but have worsened ^1^.

The acceleration of technological change also brings considerable challenges. Recent advances in the applications of artificial intelligence (AI), with the emergence of multimodal transformative models and algorithmic “agents,” add new layers of uncertainty in an already troubled world. As Srnicek ^2^ recently noted, the fundamental decisions about the development of AI are in the hands of a small elite in the highest strata of corporate and government power, but they will impact the lives of billions of people. Notably, the evolution of these technologies will enlarge the gap between decision-making power and impacts.

In the United States, the second Trump administration has been a remarkable disruptor. The kidnapping of Venezuela’s president in January 2026 by US military forces constitutes an unprecedented violation of national sovereignty, even considering the long history of US-backed and sponsored coups d’état in the Americas. The war unleashed against Iran by the United States and Israel the following month is yet another attack on international law and is part of a strategy to recompose the unipolar supremacy of the United States in the global system, collapsing the so-called “rules-based international order”, previously a cornerstone of US foreign policy ^3^.

China’s rise to the status of an economic and technological superpower capable of rivaling the United States is a central element in the current political landscape. Trumpism’s accelerationist and disruptive strategy, including threats to unilaterally impose tariffs and sanctions on countries and companies, is essentially due to China’s advance in competition for markets and innovation. China is now the main trading partner of most Latin American countries and has been expanding its presence in the region. In response, the United States increase the pressure on Latin American countries and assume a more openly interventionist posture over their “backyard” ^4^.

In this context, what future and what challenges can be expected for health systems in the so-called Latin America? To answer this question, it is necessary to highlight “structural” aspects, linked to the political trajectory and economic insertion of these countries, which provides a general understanding of the constitution of their health systems and their possibilities for development, also addressing the challenges of the conjuncture. Regarding structural dimension, we propose a schematic model that articulates recent transformations of the State and health systems, initially in more abstract terms, based on contributions from British political scientists Michael Moran ^5^ and Bob Jessop ^6^, to then observe them in the Latin American context. In this scheme, it is assumed that conflicts of interest at the national level will be arbitrated under the conventional terms and limits of the democratic game, which is not guaranteed. These issues will be addressed in the conclusion.

A problem arises at the outset: the very term “Latin America” is not unambiguous, nor is its meaning self-evident. The qualifier “Latina” refers to colonization − as in “Hispano-America” or “Ibero-America”, but the division into three “Americas” − North, South, and Central - is also common, and it should be noted that South America is home to three former non-Iberian colonies: British, Dutch, and French, the latter still with the status of “overseas department” of its center. If we add “Caribe”, the situation gets further complicated. In fact, in the universe of international intitutions such as the World Bank and the United Nations (UN) itself, we come across the acronym “LAC”, Latin America and the Caribbean, designating a geographical entity as broad and distinct as it is heterogeneous.

Accepting that the limits of such definition are largely imprecise, we argue that it is possible to speak of “Latin America” as a primarily political entity. In this case, the reference to the colonial past seems to be lesser than the contrast with the “other America”, i.e., the Anglo-Saxon America. It is not by chance that the term emerges in the mid-19th century, in the face of the aggressive territorial expansionism of the United States, notably after the war against Mexico, when the United States seized about half of Mexico’s territory ^7^. 

As Celso Furtado ^8^ pointed out, the international division of labor in the early phases of the industrial revolution would have blocked the emergence of a shared identity and, although the retraction of international trade after the 1929 crisis favored industry and the diversification of trade in the region, a Latin American proto-consciousness would only emerge in the 1950s. During this decade, the limits of industrialization by export substitution became evident and the debate on the obstacles to regional development deepened. Furtado highlights the growing relations of dependence with the United States and the entry of US capital, configuring a clear relationship of economic domination that has led to a strengthening of ties in the region ^8^.

In the meantime, the United States sponsored dozens of *coups d’état* in its southern neighbors, manipulating disputes between local oligarchies or through direct intervention. During the Cold War, the United States promoted and sustained bloody dictatorships in countries such as Chile, Uruguay, Argentina, and Brazil. It is not by chance that the wave of redemocratization in the region occurred in the period of détente of the Cold War and advanced with the implosion of the Soviet Union ^9^.

Nowadays, Latin American countries are autonomous political communities, with varied territorial dimensions and economies with different levels of complexity. They are similar in terms of high internal inequalities and differ in the greater or lesser presence of surviving indigenous populations and descendants of enslaved Africans, as well as by the greater or lesser influx of European and Asian immigrants in the 19th and 20th centuries, resulting in different forms of expression of social hierarchies. These inequalities, associated with the peripheral insertion in the international order, feed back into an unbalanced development model, dependent on and associated with internationalized big capital, sustained by political and economic elites, in general, little committed to the future of their populations.

As Darcy Ribeiro ^10^ pointed out, at precise historical moments there is the decisive rise of “anti-elites” who manage to reach positions of power, often in coalition with “authoritarian elites”, and introduce modernizing reforms. An example of this is the consolidation of labor rights in Mexico, Brazil and Argentina, respectively under Cardenas, Vargas, and Perón, in the middle of the 20th century. The health systems of these countries derive from the care and social security institutions created during in this period. Popular pressure for the extension of rights broadens the social base of reformist movements, notably from the 1950s onward − the golden age of the welfare state, also marked by the expansion of Soviet socialism and anti-colonial struggles in the so-called Third World. Reformist and popular governments in Latin America were fought with the use of political violence, *coups d’état* and the implementation of regimes of exception, promoted by sectors of the local elites, with the support or direct involvement of the United States, as already mentioned ^9^.

Welfare regimes in the Latin American follow the uneven pace of industrialization and urbanization. In countries such as Brazil, Chile, and Argentina, the first aid funds emerged at the early 20th century. Note that the boost for import substitution industrialization, starting in the 1930s and during World War II, led to the introduction of reforms in a broader set of countries, including Mexico, Venezuela, Colombia, and Peru. Smaller and poorer countries, mainly in Central America (excepting Costa Rica), generally carried out late and limited reforms ^11^.

Organized according to the medical models and practices of the central countries, Latin American health systems are equally affected by the “metamorphoses of capitalism” at the global level ^12^: geopolitical and geo-economics disputes, technological transitions and concomitant transformations of the structure of States. In contrast to eminently redistributive social policies, health care presupposes a productive complex composed of a service sector with specialized labor and a productive sector in order to supply inputs and equipment ^13^. Such characteristics make health care not only a decisive arena for the dispute of the distributive conflict, but also a frontier for the development and incorporation of complex technologies and for the accumulation of capital.

Michael Moran ^5^ introduced the concept of “Health Care State” to highlight aspects of health care that distinguish it from other welfare policies and the role played by the State in this sphere in contemporary societies. The “Health Care State” would have three inseparable faces: (1) “welfare State” aimed at social reproduction and reduction of class conflicts; (2) “capitalist industrial state”, which fosters the productive complex as a precondition for the provision of care and the accumulation of capital in the sector; and (3) “pluralist democratic state”, which manages conflicts of interest between different segments in society. Elaborated based on the experience of the central countries, the model is applicable to other contexts, with differences related, for example, to the “industrial-capitalist” face, depending on the dimensions of the production complex, local production capacity and degree of dependence on imported technology; but also, with the “pluralist democratic” face, due to different levels of inequality and social class structure in different national contexts.

Jessop ^6^ identified a recent process of transition between predominant forms of the capitalist State, from the “Keynesian national welfare state” to what he called the “Schumpeterian competitive state”, which converge with the first two “faces” of Moran’s “Health Care State”, adding a new interpretative layer: the structural constraints that shape the competitive insertion of national states in the new architecture of globalized capitalism. In this context, the State must, on the one hand, directly or indirectly sustain the demand for health goods and services through subsidies, credit, and financing, and, on the other hand, guarantee the supply of technologies, infrastructure and inputs, as well as the training of human resources. It is noteworthy that the most sophisticated technologies and resources are mostly controlled and/or owned by large transnational companies. At the same time, the State must mediate and manage numerous conflicts of interest that oppose suppliers and consumers, employers and workers, in a framework of generalized fiscal restraint, which results from the neoliberal reform agenda imposed in recent decades.

The so-called “digital transformation”, whose impact extends to all spheres and circuits of the health production chain − from industrial production to final consumption − brings a considerable set of challenges that demand management and regulatory skills and capabilities that, in practice, represent more budgetary pressure: protection and privacy of sensitive personal data deposited in immense “data lakes”; supervision of databases under private control; collection and sharing of data through mobile devices, body sensors, etc.; regulation of medical devices based on AI, including rules for access to the databases necessary for the “training” of algorithms, and, not least, the confrontation of systematic disinformation disseminated via social media.

The current model of capitalist development in health is based on the thin balance between interests linked to accumulation − present in industry and in the insurance and service sectors − and the need to provide bare minimum to society and be capable of guaranteeing the reproduction of the workforce under worsening social conditions. This minimum serves as a buffer against social conflicts and also as reinsurance for the private health insurance sector, which covers less disadvantaged segments of the population. This is a reality that imposes itself on all Latin American countries, despite the different trajectories and processes of institutional construction.

Nevertheless, the uncertainties pointed out at the beginning of this article, related to the intensification of geopolitical rivalries, the effects of the climate emergency, and the acceleration of technological change, have led to the resurgence of nationalism, religious fanaticism, and supremacist racism, which are reflected in the rise and electoral success of authoritarian movements and leaderships. Millions of refugees from the ongoing wars in the Middle East and Africa, and the normalization of massacres, as seen in Gaza, put us in front of a humanitarian crisis of gigantic proportions. More frequent climate catastrophes and the outbreak of new pandemics, for which health systems and international agencies are not adequately prepared ^1^, complete the picture.

These are real threats to the civilizational achievements of the 20th century, including the idea that the right to health and a dignified life are extended to humanity as a whole. There is no guarantee that these setbacks will be reversed in the short term, but it is certain that there will be no return to the status quo ante. Above all, there are the corrosive effects of persistent inequality and endemic violence, the growing sense of insecurity and the absence of prospects for the future, especially for the youngest.

The great challenge of Latin American health systems, at this juncture, is to sustain and give credibility to the idea of Health as a public good and a fundamental right. This will essentially depend on the “health of democracy” in the region, but the reverse is also true: the health of democracy is conditioned by the ability to guarantee decent health for its citizens.
