# Supplementary material for: Challenges faced by Latin America health systems
Source: Cad Saude Publica. 2026 Aug 10;42(Suppl 1):e00233225. doi: 10.1590/0102-311XEN233225 (PMC13460890; doi:10.1590/0102-311XEN233225)
Supplement: Translation

# Translation: Desafios dos sistemas de saúde na América Latina

**DOI:** 10.1590/0102-311XPT233225

**Affiliations:** 1 Escola Nacional de Saúde Pública Sergio Arouca, Fundação Oswaldo Cruz, Fiocruz, Rio de Janeiro, Brasil.

Este artigo tem por objetivo propor uma reflexão abrangente, ao mesmo tempo panorâmica e sintética, sobre os desafios atuais dos sistemas de saúde na América Latina. Não é tarefa simples, o momento é de extrema incerteza com relação ao futuro próximo. A emergência climática e a ameaça de novas pandemias constituem, por si sós, desafios imensos para os sistemas de saúde. À perturbação dos ecossistemas e perda de biodiversidade com suas consequências nefastas somam-se retrocessos democráticos, aumento das desigualdades, guerras e o acirramento dos conflitos geopolíticos. Pouco tempo passado desde a COVID-19, as condições políticas e econômicas que favorecem as pandemias não apenas continuam como se agravaram ^1^.

A aceleração da mudança tecnológica traz igualmente desafios consideráveis. Progressos recentes nas aplicações de inteligência artificial (IA), com o surgimento de modelos transformativos multimodais e “agentes” algorítmicos, acrescentam novas camadas de incerteza em um mundo já conturbado. Como observou recentemente Srnicek ^2^, as decisões fundamentais sobre o desenvolvimento dessas tecnologias estão em mãos de uma pequena elite nos altos escalões do poder corporativo e governamental, mas terão impacto sobre as vidas de bilhões de pessoas e, com a evolução dessas tecnologias, a divergência de escala entre poder de decisão e impactos tende a crescer.

O segundo governo de Donald Trump (Estados Unidos) tem sido um fator descomunal de disrupção. O sequestro do presidente da Venezuela, em janeiro de 2026, por forças militares estadunidenses, constitui uma violação de soberania inédita, mesmo considerando o amplo histórico de golpes de Estado apoiados e patrocinados pelos Estados Unidos na região. A guerra deflagrada contra o Irã por Estados Unidos e Israel, no mês seguinte, é mais um atentado contra o direito internacional e faz parte de uma estratégia de recomposição da supremacia unipolar dos Estados Unidos no sistema mundial, colapsando a chamada “ordem internacional baseada em regras”, antes um pilar da política externa estadunidense ^3^.

A ascensão da China à condição de superpotência econômica e tecnológica capaz de rivalizar com os Estados Unidos é um elemento central nesse quadro. A estratégia aceleracionista e disruptiva do *trumpismo*, incluindo ameaças de imposição unilateral de tarifas e sanções a países e empresas, deve-se essencialmente ao avanço chinês na competição por mercados e inovação. O gigante asiático é hoje o principal parceiro comercial da maioria dos países latino-americanos e vem ampliando a sua presença na região. Em resposta, os norte-americanos aumentam a pressão sobre os governos locais e assumem uma postura mais abertamente intervencionista sobre seu “quintal” ^4^.

Nesse contexto, qual futuro e quais desafios para os sistemas de saúde na chamada América Latina? Para responder a essa questão é preciso, por um lado, destacar aspectos de natureza “estrutural”, ligados à trajetória política e inserção econômica desses países, que permitem compreender, em termos gerais, a constituição de seus sistemas de saúde e suas possibilidades de desenvolvimento, mas é preciso considerar também os desafios da conjuntura. Com relação à dimensão estrutural, propomos um modelo esquemático que articula transformações recentes do Estado e dos sistemas de saúde, inicialmente em termos mais abstratos, com base em contribuições de dois autores britânicos, os politólogos Michael Moran ^5^ e Bob Jessop ^6^, para, então, observá-los no contexto latino-americano. Nesse esquema está pressuposto que os conflitos de interesse em âmbito nacional serão arbitrados nos termos e limites convencionais do jogo democrático, o que não está garantido. Trataremos desses temas na conclusão.

Um problema se impõe de início: o próprio termo “América Latina” não é inequívoco, nem seu significado autoevidente. O qualificativo “Latina” remete à colonização − como em “Hispano-América” ou “Ibero-América”, mas é também corrente a divisão em três “Américas” − Norte, Sul e Central − e note-se que a América do Sul abriga três ex-colônias não ibéricas: britânica, holandesa e francesa, esta última ainda com status de “departamento ultramarino” da metrópole. Se acrescentarmos o “Caribe”, a situação se complica. Com efeito, no universo das agências internacionais como o Banco Mundial e a própria ONU, nos deparamos com a sigla “LAC”, *Latin America and the Caribbean*, designando um ente geográfico tão amplo e distinto quanto heterogêneo.

Aceitando que seus limites são necessariamente imprecisos, argumentamos que é possível falar de “América Latina” como ente sobretudo político. Mais decisivo, neste caso, que a referência ao passado colonial parece ser o contraste com a “outra América”, anglo-saxã. Não por acaso o termo surge em meados do Século XIX, face ao agressivo expansionismo territorial dos Estados Unidos, notadamente após a guerra contra o México, quando os Estados Unidos tomaram cerca da metade do território daquele país ^7^.

Como assinalou Celso Furtado ^8^, o regime de divisão internacional do trabalho nas primeiras fases da revolução industrial teria bloqueado o surgimento de uma identidade compartilhada e, embora a retração do comércio internacional pós-crise de 1929 tenha favorecido a indústria e a diversificação do comércio na região, uma proto-consciência latino-americana surgiria somente na década de 1950, quando se evidenciaram os limites da industrialização por substituição de exportações e se aprofundou o debate sobre os obstáculos ao desenvolvimento regional. Furtado ressalta as relações crescentes de dependência com os Estados Unidos e a penetração de capitais norte-americanos, configurando uma clara relação de dominação econômica que propiciou um estreitamento de vínculos na região ^8^.

Nesse interregno, os Estados Unidos patrocinaram dezenas de golpes de Estado nos vizinhos do Sul, manipulando disputas entre oligarquias locais ou mesmo intervindo diretamente. No período da Guerra Fria, promoveram e sustentaram ditaduras sangrentas em países como Chile, Uruguai, Argentina e Brasil. Não por acaso, a onda de redemocratização na região ocorreu no período de distensão da Guerra Fria e avançou com a implosão do bloco soviético ^9^.

As repúblicas da América Latina atual são comunidades políticas autônomas, com dimensões territoriais variadas e economias com níveis diferenciados de complexidade. Assemelham-se por elevadas desigualdades internas e diferenciam-se pela maior ou menor presença de populações autóctones sobreviventes e descendentes de africanos escravizados, bem como pelo afluxo maior ou menor de imigrantes europeus e asiáticos nos séculos XIX e XX, resultando em formas distintas de expressão das hierarquias sociais. Essas desigualdades, associadas à inserção periférica na ordem internacional, retroalimentam um modelo de desenvolvimento desequilibrado, dependente e associado ao grande capital internacionalizado, sustentado por elites políticas e econômicas, em geral, pouco compromissadas com os destinos de suas populações.

Como apontou Darcy Ribeiro ^10^, há o fator decisivo da emergência, em momentos históricos precisos, de “antielites” que logram alcançar posições de poder, frequentemente em coalizão com “elites autoritárias”, e introduzem reformas modernizantes. Exemplo disso é a consolidação dos direitos trabalhistas no México, Brasil e Argentina, respectivamente sob Cardenas, Vargas e Perón, em meados do século passado. Os sistemas de saúde desses países derivam das instituições assistenciais e previdenciárias criadas nesse processo. A pressão popular pela extensão de direitos amplia a base social dos movimentos reformistas, notadamente a partir da década de 1950 − a era dourada do Estado assistencial, marcada igualmente pela expansão do socialismo soviético e pelas lutas anticoloniais no chamado Terceiro Mundo. Governos reformistas e populares na América Latina foram combatidos com recurso à violência política, golpes de Estado e implantação de regimes de exceção, promovidos por setores das elites locais, com o apoio ou o envolvimento direto dos Estados Unidos, como já mencionado ^9^.

Os regimes de bem-estar na região acompanham o passo desigual dos processos de industrialização e urbanização. Em países como Brasil, Chile e Argentina, as primeiras caixas de assistência surgiram no início do século XX, mas o impulso à substituição de importações, a partir dos anos 1930 e durante a Segunda Guerra, ensejou a introdução de reformas em um conjunto mais amplo de países, incluindo México, Venezuela, Colômbia e Peru. Países menores e mais pobres, principalmente na América Central (com exceção da Costa Rica), realizaram, em geral, reformas tardias e limitadas ^11^.

Organizados conforme modelos e práticas médicas dos países centrais, os sistemas de saúde latino-americanos são igualmente afetados pelas “metamorfoses do capitalismo” em nível global ^12^: disputas geopolíticas e geoeconômicas, transições tecnológicas e transformações concomitantes da estrutura dos Estados. Em contraste com políticas sociais eminentemente redistributivas, a atenção à saúde pressupõe um complexo produtivo composto por um setor de serviços intensivo em mão de obra especializada e um setor de produção de insumos e equipamentos ^13^. Tais características fazem da saúde não somente uma arena decisiva de disputa do conflito distributivo, mas, também, fronteira para o desenvolvimento e incorporação de tecnologias complexas e para a acumulação de capital.

Michael Moran ^5^ introduziu o conceito de *Health Care State* para destacar aspectos da assistência à saúde que a distinguem das demais políticas de bem-estar e o papel desempenhado pelo Estado neste âmbito, nas sociedades contemporâneas. O “Estado de Assistência à Saúde” apresentaria três faces indissociáveis: (1) “Estado de bem-estar” voltado para a reprodução social e redução dos conflitos de classe; (2) “Estado industrial capitalista”, que fomenta o complexo produtivo como precondição da prestação de serviços assistenciais e da acumulação de capital no setor; e (3) “Estado democrático pluralista”, que administra os conflitos de interesse entre segmentos distintos na sociedade. Elaborado com base na experiência dos países centrais, o modelo é aplicável a outros contextos, com diferenças relacionadas, por exemplo, com a face “industrial-capitalista”, em função das dimensões do complexo produtivo, capacidade de produção local e grau de dependência de tecnologia importada; mas, também, com a face “democrático-pluralista”, em razão de níveis de desigualdade e estrutura de classes sociais distintos nos diferentes contextos nacionais.

Jessop ^6^ identificou um processo recente de transição entre formas predominantes do Estado capitalista, do “Estado nacional de bem-estar keynesiano” para o que chamou de “Estado competitivo schumpeteriano”, que convergem com as duas primeiras “faces” do *Health Care State* de Moran ^5^, acrescentando uma nova camada interpretativa: os constrangimentos estruturais que moldam a inserção competitiva dos Estados nacionais na nova arquitetura do capitalismo globalizado. Nesse contexto, cabe ao Estado, por um lado, sustentar a demanda por bens e serviços de saúde por meio de subsídios, crédito e financiamento, direto e/ou indireto e, por outro lado, garantir a oferta de tecnologias, infraestrutura e insumos, bem como a formação de recursos humanos, neste caso, em um contexto no qual as tecnologias e recursos mais sofisticados são majoritariamente controlados e/ou propriedade de grandes empresas transnacionais. Ao mesmo tempo, o Estado deve mediar e gerenciar inúmeros conflitos de interesse que opõem fornecedores e consumidores, empregadores e trabalhadores, em um quadro de restrição fiscal generalizada, que resulta da agenda de reformas neoliberais impostas nas últimas décadas.

A chamada “transformação digital”, cujo impacto se estende a todas as esferas e circuitos da cadeia de produção da saúde, da produção industrial ao consumo final, traz um conjunto considerável de desafios que demandam competências e capacidades de gestão e regulação que, na prática, significam mais pressão orçamentária: proteção e privacidade de dados pessoais sensíveis depositados em imensos “lagos de dados”; supervisão de bases de dados sob controle privado; coleta e compartilhamento de dados por meio de dispositivos móveis, sensores corporais etc; regulação de dispositivos médicos baseados em IA, incluindo regras de acesso às bases de dados necessárias para o “treinamento” de algoritmos e, não menos importante, o enfrentamento da desinformação sistemática veiculada por intermédio de redes digitais.

O atual modelo de desenvolvimento capitalista na saúde se sustenta no equilíbrio precário entre, de um lado, interesses ligados à acumulação, presentes na indústria e nos setores de seguros e serviços, e, de outro lado, a necessidade de prover um mínimo social capaz de garantir a reprodução da força de trabalho sob crescente precarização. Tal mínimo serve como amortecedor de conflitos sociais e, também, como resseguro para o setor privado de planos de saúde, que recobre segmentos menos desfavorecidos da população. É uma realidade que se impõe ao conjunto dos países latino-americanos, apesar das trajetórias e processos de construção institucional distintos.

Por outro lado, as incertezas apontadas no início deste artigo, relacionadas com o acirramento das rivalidades geopolíticas, com os efeitos da emergência climática e com a aceleração da mudança tecnológica, têm levado ao recrudescimento dos nacionalismos, do fanatismo religioso e do racismo supremacista, que se refletem na ascensão e sucesso eleitoral de movimentos e lideranças de caráter autoritário. Milhões de refugiados das guerras em curso, no Oriente Médio e na África, e a normalização de massacres, como visto em Gaza, nos colocam frente a uma crise humanitária de proporções gigantescas. Catástrofes climáticas mais frequentes e a eclosão de novas pandemias, para as quais os sistemas de saúde e agências internacionais não se encontram adequadamente preparados ^1^, completam o quadro.

São ameaças reais às conquistas civilizatórias do século passado, incluindo a ideia de que o direito à saúde e a uma vida digna são extensivas ao conjunto da humanidade. Não há garantia de que esses retrocessos sejam revertidos no curto prazo, mas é certo que não haverá retorno ao *status quo ante*. Sobretudo, há os efeitos corrosivos da desigualdade persistente e da violência endêmica, da crescente sensação de insegurança e da ausência de perspectivas de futuro, principalmente para os mais jovens.

O grande desafio dos sistemas de saúde latino-americanos, nessa conjuntura, é sustentar e dar credibilidade à ideia da Saúde como bem público e direito fundamental. Isso vai depender essencialmente da “saúde da democracia” na região, mas o inverso também é verdadeiro: a saúde da democracia está condicionada pela capacidade de garantir saúde digna para os seus cidadãos.
